# Supplementary material for: Complexome profiling of the Chlamydomonas psb28 mutant reveals TEF5 as an early PSII assembly factor
Source: Plant Cell. 2025 Mar 17;37(6):koaf055. doi: 10.1093/plcell/koaf055 (PMC12164590; doi:10.1093/plcell/koaf055)
Supplement: koaf055_Supplementary_Data [file koaf055_supplementary_data.zip › psb28_interactive.zip!/plots/intensity/qualityPlots/peptides/_Cre02.g094250.t1.1.html]

### Cre02.g094250.t1.1

Trivial name:   
SeqCoverage [median]: 45.20  
Mass: 43.93 kDa  
  
 

| Sequence | MissedCleavage | Mass [Da] | wtIntensity | muIntensity | Score |
| --- | --- | --- | --- | --- | --- |
| AAGSGPGGAAAR | 0 | 941.47 | 5567.20 | 2460.26 | 128.12 |
| AALSLPLPR | 0 | 936.58 | 33448.67 | 49588.70 | 129.40 |
| AGAIGGGPGGGR | 0 | 925.47 | 8648.87 | 10209.13 | 130.28 |
| AVAWQR | 0 | 729.39 | 411.39 | 0.00 | 74.85 |
| FLQTYK | 0 | 798.43 | 1366.24 | 2063.57 | 97.60 |
| IGATVVTYPLIVVK | 0 | 1471.90 | 12756.40 | 0.00 | 152.07 |
| IQGYQSLPGAK | 0 | 1160.62 | 22840.18 | 17050.47 | 139.39 |
| LPAPPPAMPK | 0 | 1017.57 | 16216.63 | 11867.09 | 93.26 |
| LSATEVFLAGALAK | 0 | 1389.79 | 11041.92 | 0.00 | 207.34 |
| LVARPVAPAVK | 1 | 1119.71 | 19577.49 | 9211.99 | 67.73 |
| LVARPVAPAVKAPSAAG | 2 | 1573.93 | 692.18 | 1056.70 | 43.79 |
| NEGLGGFFK | 0 | 967.48 | 5595.72 | 2284.76 | 87.70 |
| PAALLSAADVK | 0 | 1054.60 | 368.92 | 1655.12 | 98.03 |
| PVSTWSVISETAR | 0 | 1431.74 | 414.75 | 1401.51 | 116.77 |
| QAELEAEEHRETR | 1 | 1596.75 | 11001.37 | 3944.77 | 110.38 |
| SGPAAPLSGPSK | 0 | 1067.56 | 4397.30 | 4448.29 | 98.94 |
| TIYTLQALSTGSGDAAPSVPTDDNK | 0 | 2521.22 | 1641.02 | 7283.42 | 76.89 |
|  |  |  | 155986.3 | 124525.8 |  |
